# Prevalence and associated factors of irritable bowel syndrome among medical students in Ethiopia: a cross-sectional study

**DOI:** 10.1186/s12876-025-04415-8

**Published:** 2025-11-26

**Authors:** Elias Tafesse Yeshitila, Nanati Jemal Aliye, Abdulsemed Mohammed Nur, Mengistu Yilma

**Affiliations:** 1 Department of Medicine, Addis Ababa University, Addis Ababa, Ethiopia; 2 Department of Public Health, Addis Ababa University, Addis Ababa, Ethiopia

**Keywords:** Irritable bowel syndrome, Medical students, Ethiopia, Anxiety, Rome IV, Cross-Sectional study

## Abstract

**Background:**

Irritable bowel syndrome (IBS) is a common disorder of gut-brain interaction, yet data from sub-Saharan Africa remain limited. In Ethiopia, evidence on IBS among medical students is particularly limited, despite this group’s high exposure to academic and psychosocial stressors.

**Objectives:**

To estimate the prevalence of IBS among Ethiopian medical students and to examine its demographic, lifestyle, and psychological correlates.

**Methods:**

A cross-sectional analytical study was conducted between 1 June and 31 August 2024 among undergraduate medical students from eleven Ethiopian universities. Participants completed a self-administered online questionnaire incorporating the Rome IV criteria for IBS and the Hospital Anxiety and Depression Scale (HADS). Descriptive statistics summarized participant characteristics. A mixed-effects logistic regression with a random intercept for university was used to assess associations between IBS and prespecified predictors (sex, anxiety, and depression). Wald odds ratios (ORs) with 95% confidence intervals (CIs) were reported. Sensitivity analyses used Firth penalised logistic regression, exclusion of small clusters, and binary recoding of HADS categories. Model discrimination and calibration were assessed using the area under the receiver operating characteristic curve (AUC) and calibration slope and intercept.

**Results:**

A total of 290 medical students participated (mean age = 23 years). Twenty students met the Rome IV criteria for IBS, corresponding to a prevalence of 6.9% (95% CI 4.2–10.4). In the adjusted model, anxiety classified as “case” was associated with higher odds of IBS (OR 7.25, 95% CI 1.83–28.72; *p* < 0.01). Borderline anxiety, depression (borderline or case), and female sex were not statistically significant. Sensitivity analyses produced similar estimates. The model showed acceptable discrimination (AUC = 0.78; optimism-corrected AUC = 0.73) and good calibration (intercept = 0.00; slope = 1.00).

**Conclusions:**

The prevalence of IBS among Ethiopian medical students was 6.9%. Anxiety was statistically associated with IBS, whereas depression and sex were not. The findings highlight the presence of IBS and psychological correlates among this population. Broader longitudinal research could further clarify the epidemiology and psychosocial factors related to IBS in Ethiopian medical students.

**Supplementary Information:**

The online version contains supplementary material available at 10.1186/s12876-025-04415-8.

## Introduction

Irritable bowel syndrome (IBS) is among the disorders of gut-brain interaction (also known as functional disorders of the gastrointestinal system) manifesting with episodic pain and altered bowel habits with no detectable evidence of structural abnormality of the gastrointestinal tract (GIT) [1, 2].

IBS is formally defined using the Rome IV criteria as history of recurrent abdominal pain that occurs at least one day a week (on average) in the last 3 months, associated with at least two of the following: pain related to defecation, a change in the frequency of stool, or a change in the appearance of stool. The symptoms must have started at least 6 months before diagnosis for IBS to be defined [3, 4].

Although not fatal, IBS can substantially reduce health-related quality of life, with impacts comparable to other chronic conditions and has a significant economic impact [5]. IBS has no cure, but its symptoms are treatable with a combination of lifestyle, dietary, and pharmacologic methods [6]. To date, several factors have been consistently associated with an increased likelihood of developing IBS. These include family history, female sex, smoking, consumption of canned food or fast food, anxiety, depression and psychological stress [2, 7–12].

Information on the worldwide prevalence of IBS is limited. The few studies on this topic have used different criteria to define IBS in participants, which has led to a wide discrepancy in the results of the studies compared with one another [2]. For example, studies that used the Rome III criteria reported a significantly higher worldwide prevalence of 10.1% than those that used the Rome IV criteria, which reported a prevalence of 3.8% [2].

In contrast, studies indicate significantly higher rates of IBS prevalence in medical students worldwide, ranging from 9.3% to 35.5%. Studies from low- and middle-income countries, including Egypt, Nigeria, Benin, and Tunisia, reported that the prevalence of IBS among medical students ranged from 7.6% to 30% [13–16]. These studies revealed varied associations with sex, diet, and psychological factors, highlighting inconsistencies in IBS patterns across different African populations.

Despite the apparent burden, there is currently no published study on the prevalence of irritable bowel syndrome (IBS) in Ethiopia. Additionally, aside from a recent preprint that evaluated the prevalence of IBS among clinical medical students at a single university in Ethiopia [17], there is little research on the prevalence of IBS at the national level. Information on the predictors of IBS in Ethiopian medical students is also unavailable. This represents a significant gap in both clinical knowledge and policy-relevant data. Medical students face high academic stress, irregular sleep patterns, and frequent mental strain. These conditions increase the risk of gut-brain disorders such as IBS. IBS can also affect students’ academic performance, mental health, and future professional functioning. Accordingly, identifying its predictors and addressing its impact is essential.

## Objectives

This study therefore aims to do the following:

1. Determine the prevalence rate of IBS in Ethiopian medical students and.
2. Determine the predictors of IBS in Ethiopian medical students.

## Methods

### Study Design and Setting

This was a cross-sectional analytical study conducted among undergraduate medical students enrolled in eleven accredited public and private universities across Ethiopia. The source population comprised all students registered in these universities’ undergraduate medical programs during the 2023–2024 academic year. A self-administered online questionnaire, prepared in English using Google Forms, was distributed between 1 June and 31 August 2024. The survey link was shared through official class group social media channels managed by student representatives. Participation was voluntary and required internet access and engagement with these platforms. Informed consent was obtained electronically at the start of the questionnaire after providing an explanation of the study’s purpose.

### Participants

Participants were undergraduate medical students from several public and private medical colleges. Students were eligible if they were currently enrolled in any year of their university’s undergraduate medical program and provided informed consent.

The exclusion criteria were the presence of bloody stools in the past 6 months and history of colon cancer in self or a first-degree relative. The exclusion criteria were embedded in the Google Form, and the Google Form automatically excluded participants who possessed these criteria.

### Variables

The primary outcome variable was *the presence or absence of irritable bowel syndrome*, as determined by the Rome IV IBS questions embedded in the survey.

The independent variables were all categorical and included:

1. Demographics: sex (male/female), age group (≤ 20, 21–30, > 30), marital status (unmarried/married), and year of study (Years I–IV, internship).
2. Lifestyle behaviors: alcohol use (yes/never), smoking (yes/never), self-reported physical activity (yes/never), self-reported fast-food consumption (including French fries, erteb, and sambusa; yes/never), and sleep duration on most days (< 7 h/≥7 h). For lifestyle items, “yes” encompassed responses such as “sometimes” and “regularly.”
3. Health indicators: family history of IBS (yes/no), allergies (yes/no), chronic illness (yes/no), GPA category (< 3.5 vs. 3.5–4.0), and accommodation (dormitory/with family/off-campus away from family).
4. Psychological factors: anxiety and depression were measured using the Hospital Anxiety and Depression Scale (HADS), a 14-item instrument (7 anxiety, 7 depression), each item scored 0–3. Subscale totals were categorized as normal (0–7), borderline (8–10), or case (11–21) [18–20].

The levels of all variables were derived from clinically relevant cutoffs.

### Data Sources and Measurement

Data were collected through a self-administered questionnaire, which included standardized questions on IBS symptoms, lifestyle habits, and mental health. Where possible, questions were adapted from validated tools. Prior to the main data collection, a pilot test was conducted on 17 medical students to ensure clarity and face validity, and only typographical corrections were deemed necessary after testing.

### Study size

A total of 288 medical students were planned to be included. The sample size was calculated via the following formula:

$$\mathrm n=\left(Z\alpha/2\right)^{2\ast}\mathrm p\left(1-\mathrm p\right)/\mathrm d^2$$

where n = sample size, Zα/2 = Z value at (α = 0.05) = 1.96, p = prevalence = 23% (estimated from the study conducted at the University of Gondar [17]). The proportion of nonoccurrence events to be studied was calculated as follows: 1-*p* = 1-0.23.23.23.23.23 = 0.77 d = margin of error (precision) = 0.05. A non-response rate of 5% was expected, resulting in a minimum sample size of 288.

### Bias

To minimize selection bias associated with the voluntary online questionnaire, medical students were recruited from multiple academic years and across different universities. Moreover, designated investigators at each institution actively encouraged participation, aiming to reduce the risk of preferential response from individuals with heightened health awareness or preexisting IBS symptoms.

### Statistical methods

#### All analyses were performed via R version 4.4.1

Frequencies and percentages were used to describe participant characteristics and IBS prevalence. The variables are summarized by IBS status, as shown in the table below.

We examined the association between IBS (binary) and key predictors using a mixed-effects logistic regression with a random intercept for university (11 universities). Models were fit with glmer() (lme4). For the primary analysis we reported Wald odds ratios (ORs) with 95% Wald confidence intervals and Wald p-values for fixed effects. Given the limited number of IBS events, we prespecified a parsimonious model including anxiety, depression, and sex (selected based on prior literature [21, 22]), while describing all other variables descriptively.

#### Sensitivity analysis

We prespecified three checks: (i) refitted the mixed-effects model after excluding universities with fewer than five respondents, (ii) fitted a Firth penalized logistic regression with the same predictors to assess small-sample bias and separation, and (iii) recoded HADS anxiety and depression as binary variables (borderline + case vs. normal) and refitted the mixed-effects model. Mixed-effects specifications retained a random intercept for university.

#### Model Performance and Evaluation

Discrimination was assessed using ROC curves based on marginal predicted probabilities from the mixed-effects model. The area under the ROC curve (AUC) was computed with the pROC package, and a bootstrap 95% CI (2,000 resamples) was obtained. To adjust for potential overfitting, we estimated optimism via a cluster bootstrap resampling of universities and reported the optimism-corrected AUC. An AUC greater than 0.7 was considered acceptable, as recommended in the literature [23].

Calibration was evaluated by logistic recalibration, reporting the calibration intercept (calibration-in-the-large) and calibration slope with Wald 95% CIs (ideal: intercept = 0, slope = 1). We also produced a calibration plot: a smoothed reliability curve (binomial GLM with a spline for the predicted probability) overlaid with decile-wise observed event rates and a 45° reference line. Discrimination was summarized with the AUC (pROC) and bootstrap 95% CI (2,000 resamples), and an optimism-corrected AUC was obtained via a cluster bootstrap resampling of universities.

#### Handling of Missing Data

The final dataset contained no missing data on the included variables, as the submission of the questionnaires was completed once a participant finished all the questions and submitted them. Therefore, no imputation or exclusion procedures were needed.

#### Ethical considerations

This study was conducted in accordance with the ethical standards of the institutional research committee and with the Helsinki Declaration. Ethical approval was obtained from Addis Ababa University Internal Medicine Department Review Board, and informed consent was obtained from all participants prior to data collection. Informed consent was obtained electronically at the start of the questionnaire after providing an explanation of the study’s purpose.

## Results

A total of 290 medical students participated in the study (completed the questionnaire). Among these students, 20 (6.9%) met the criteria for irritable bowel syndrome (IBS), whereas 270 (93.1%) did not.

### Baseline characteristics

Table 1 outlines the baseline characteristics of the sample by IBS status. Among the participants without IBS, 104 (38.5%) were male, and 166 (61.5%) were female; among those with IBS, 4 (20.0%) were male, and 16 (80.0%) were female. The majority of participants were aged between 21 and 30 years, comprising 207 (76.7%) in the non-IBS group and 17 (85.0%) in the IBS group. Overall, only 5 participants were aged over 30 years, none of whom had IBS.

**Table 1:** Baseline characteristics of undergraduate medical students in Ethiopia by irritable bowel syndrome (IBS) status (*n* = 290). The table presents sociodemographic, lifestyle, and psychosocial characteristics of participants stratified by IBS status. Percentages are shown within each outcome group. anxiety and depression were assessed using the hospital anxiety and depression scale (HADS), and IBS status was determined using the Rome IV criteria. The sample included students from eleven universities

Variable	Category	No IBS^†^ (*n* = 270, %)	IBS (*n* = 20, %)
Sex	Male	104 (38.5%)	4 (20.0%)
	Female	166 (61.5%)	16 (80.0%)
Age	< 21	58 (21.5%)	3 (15.0%)
	21–30	207 (76.7%)	17 (85.0%)
	> 31	5 (1.9%)	0 (0.0%)
Academic Year	Year I	77 (28.5%)	7 (35.0%)
	Year II	28 (10.4%)	4 (20.0%)
	Year III	33 (12.2%)	3 (15.0%)
	Year IV	100 (37.0%)	4 (20.0%)
	Internship	32 (11.9%)	2 (10.0%)
Marital Status	Unmarried	252 (93.3%)	19 (95.0%)
	Married	18 (6.7%)	1 (5.0%)
Alcohol Use	Never	168 (62.2%)	15 (75.0%)
	Yes	102 (37.8%)	5 (25.0%)
Cigarette Use	Never	260 (96.3%)	19 (95.0%)
	Yes	10 (3.7%)	1 (5.0%)
Exercise	Not regularly	243 (90.0%)	19 (95.0%)
	Regularly	27 (10.0%)	1 (5.0%)
University	Addis Ababa University	111 (41.1%)	9 (45.0%)
	Arsi University	52 (19.3%)	3 (15.0%)
	Gondar University	23 (8.5%)	1 (5.0%)
	Hawassa University	4 (1.5%)	0 (0.0%)
	Hayat Medical College	46 (17.0%)	4 (20.0%)
	Jimma University	6 (2.2%)	3 (15.0%)
	Myungsung Medical College	16 (5.9%)	0 (0.0%)
	Sante Medical College	9 (3.3%)	0 (0.0%)
	Wachamo University	1 (0.4%)	0 (0.0%)
	Mizan Tepi University	1 (0.4%)	0 (0.0%)
	Ambo University	1 (0.4%)	0 (0.0%)
Fast Food	Not regularly	213 (78.9%)	17 (85.0%)
	Regularly	57 (21.1%)	3 (15.0%)
Sleep	≥ 7 h	113 (41.9%)	11 (55.0%)
	< 7 h	157 (58.1%)	9 (45.0%)
Family History of IBS	No	251 (93.0%)	16 (80.0%)
	Yes	19 (7.0%)	4 (20.0%)
Anxiety	Absent	147 (54.4%)	4 (20.0%)
	Borderline	51 (18.9%)	3 (15.0%)
	Present	72 (26.7%)	13 (65.0%)
Depression	Absent	175 (64.8%)	8 (40.0%)
	Borderline	47 (17.4%)	8 (40.0%)
	Present	48 (17.8%)	4 (20.0%)
Allergy	No	240 (88.9%)	14 (70.0%)
	Yes	30 (11.1%)	6 (30.0%)
GPA (Grade Point Average)	< 3.5	137 (50.7%)	12 (60.0%)
	3.5–4.0	133 (49.3%)	8 (40.0%)
Chronic Illness	No	242 (89.6%)	16 (80.0%)
	Yes	28 (10.4%)	4 (20.0%)
Accommodation	With family	93 (34.4%)	9 (45.0%)
	Dormitory	161 (59.6%)	9 (45.0%)
	Neither	16 (5.9%)	2 (10.0%)

^†^IBS refers to irritable bowel syndrome

Medical students from all eleven universities participated, but Addis Ababa University had a plurality, making up 41% of the non-IBS group and 45% of the IBS group. There were three universities with less than 5 participants in the study (Ambo University, Mizan Tepi University, and Wachamo University). See Supplementary Fig. 1 in the Supplementary Materials for more details.

A notably higher proportion of students with IBS reported a family history of the condition (20.0% vs. 7.0%). Marked differences were also observed for psychological symptoms: anxiety was present in 65.0% of IBS participants compared with 26.7% of non-IBS participants, while borderline anxiety was reported by 15.0% and 18.9%, respectively. Depression showed a similar trend, with borderline symptoms reported by 40.0% of those with IBS versus 17.4% of those without, and depressive cases reported by 20.0% versus 17.8%. Allergies (30.0% vs. 11.1%) and chronic illnesses (20.0% vs. 10.4%) were likewise more frequently reported among participants with IBS.

### Inferential analysis

We fitted a mixed-effects logistic regression model with a random intercept for university to examine the associations of depression, anxiety, and sex with irritable bowel syndrome (IBS), using Hospital Anxiety and Depression Scale (HADS) categories (normal, borderline, case). The random-intercept variance was estimated at the boundary (τ² ≈ 0; intraclass correlation coefficient ≈ 0), indicating negligible between-university variation. Given the sparse and uneven distribution of cases across institutions, between-university heterogeneity could not be estimated with precision; therefore, the fixed-effect estimates are comparable to those from an ordinary logistic regression model.

In the adjusted model, students with anxiety (case) had significantly higher odds of IBS compared with those in the normal category (adjusted OR = 7.25; 95% CI 1.83–28.72; *p* < 0.01). The association for borderline anxiety was not statistically significant (adjusted OR = 1.80; 95% CI 0.37–8.80; *p* = 0.47). Similarly, neither borderline depression (adjusted OR = 1.77; 95% CI 0.55–5.71; *p* = 0.34) nor depression (case) (adjusted OR = 0.47; 95% CI 0.11–1.98; *p* = 0.30) showed significant associations with IBS. The adjusted OR < 1 for depression likely reflects small-sample variability, not a true protective effect. Female sex was also not significantly associated with IBS (adjusted OR = 1.67; 95% CI 0.52–5.42; *p* = 0.40).

Unadjusted models suggested a similar pattern, with higher crude odds of IBS for borderline depression (unadjusted OR = 3.72; 95% CI 1.33–10.45) and anxiety (case) (unadjusted OR = 6.64; 95% CI 2.09–21.07), although these effects were attenuated after adjustment. Full unadjusted and adjusted results are presented in Table 2.

**Table 2:** Associations of depression, anxiety, and sex with irritable bowel syndrome (IBS) among undergraduate medical students in Ethiopia. Odds ratios (ORs) and 95% confidence intervals (CIs) were estimated using mixed-effects logistic regression models with a random intercept for university to account for clustering across 11 institutions. Unadjusted ORs were derived from single-predictor mixed-effects models, while adjusted ORs included all predictors simultaneously. Anxiety (case) remained significantly associated with higher odds of IBS after adjustment (OR = 7.25; 95% CI 1.83–28.72; *p* < 0.01)

Variable	Category	Unadjusted OR^¶^	Unadjusted 95% CI^§^	Adjusted OR^‡¶^	Adjusted 95% CI^§^	*p*-value	
Depression^†^	Borderline depression	3.72	(1.33, 10.45)	1.77	(0.55, 5.71)	0.34	
	Depression (case)	1.82	(0.53, 6.31)	0.47	(0.11, 1.98)	0.30	
Anxiety^†^	Borderline anxiety	2.16	(0.47, 9.99)	1.80	(0.37, 8.80)	0.47	
	Anxiety (case)	6.64	(2.09, 21.07)	7.25	(1.83, 28.72)	< 0.01*	
Sex	Female (vs. Male)	2.51	(0.82, 7.70)	1.67	(0.52, 5.42)	0.40	

*Statistically significant p-values (< 0.05) are indicated with an asterisk (*)

† Reference categories: no depression; no anxiety; male sex

‡ Mixed-effects logistic regression adjusted for depression, anxiety, and sex, with a random intercept for university; 11 clusters (universities), cluster sizes ranged from 1 to 120

¶ OR = odds ratio

^§^ CI = confidence interval

Sensitivity analysis

Estimates were similar to the primary analysis. Anxiety (case) remained significantly associated with IBS (OR 7.18; 95% CI 1.77–29.10; *p* = 0.006). Borderline anxiety (OR 1.85; 95% CI 0.38–9.03; *p* = 0.446), borderline depression (OR 1.80; 95% CI 0.55–5.87; *p* = 0.331), depression (case) (OR 0.46; 95% CI 0.11–1.99; *p* = 0.299), and female sex (OR 1.61; 95% CI 0.49–5.25; *p* = 0.433) were not statistically significant. The central finding of a strong association between anxiety and IBS was robust in a sensitivity analysis that removed universities with fewer than five respondents, with effect sizes closely mirroring the primary model. This consistency suggests that including the three small university clusters did not significantly affect the results.

As a small-sample bias sensitivity, Firth penalized logistic regression produced consistent inferences: anxiety (case) remained significant (OR 6.68; 95% CI 1.84–27.11; *p* < 0.01), whereas borderline anxiety (OR 1.89; 95% CI 0.39–8.41; *p* = 0.41), borderline depression (OR 1.75; 95% CI 0.56–5.52; *p* = 0.33), depression (case) (OR 0.49; 95% CI 0.12–1.96; *p* = 0.31), and female sex (OR 1.54; 95% CI 0.54–5.27; *p* = 0.44) were not significant. (ROC/AUC was not computed for the Firth model because, after complete-case filtering for the Firth fit, one outcome class was absent; performance metrics are therefore reported for the primary mixed-effects model.)

When HADS anxiety categories were collapsed (borderline + case vs. normal), the association just missed conventional significance (OR 3.49; 95% CI 0.97–12.54; *p* = 0.055), suggesting either limited power to detect an effect with the merged coding or that borderline anxiety contributes little to the association observed for cases.

In the primary categorical model, the adjusted OR for depression (case) was below 1 but not statistically significant, indicating no clear evidence of an inverse association. When HADS-D categories were collapsed (borderline + case vs. normal), depression was not associated with IBS (OR = 1.46; 95% CI 0.51–4.21; *p* = 0.482).

### Model evaluation

**Fig. 1:**
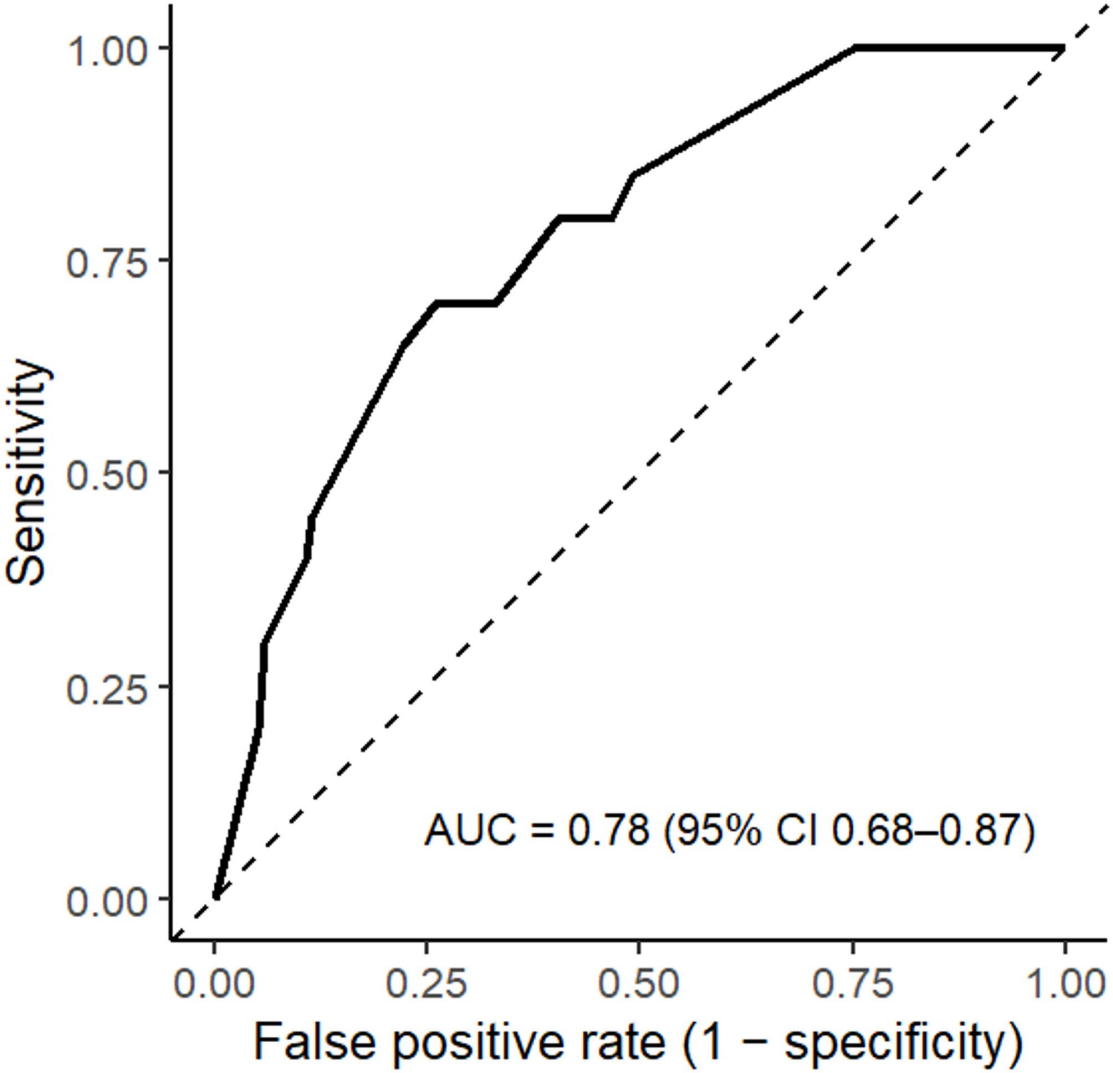
Receiver operating characteristic (ROC) curve for the mixed-effects logistic regression model predicting irritable bowel syndrome (IBS) among Ethiopian medical students. The model included anxiety, depression, and sex as fixed-effect predictors, with a random intercept for university to account for clustering. The area under the curve (AUC) was 0.78 (95% CI 0.68–0.87), indicating acceptable model discrimination between students with and without IBS. Logistic recalibration indicated good calibration: slope = 1.00 (95% CI 0.52–1.48) and intercept = 0.00 (95% CI − 0.47–0.47), i.e., predicted risks showed neither systematic under/over-dispersion (slope ≈ 1) nor overall miscalibration-in-the-large (intercept ≈ 0). The calibration plot is shown in Fig. 2

Model discrimination for the mixed-effects model was AUC = 0.78 (95% CI: 0.68–0.87) on the training data. Using a cluster bootstrap (resampling universities), the estimated mean optimism was 0.05, yielding an optimism-corrected AUC of 0.73 (95% CI 0.51–0.80), consistent with fair–acceptable discrimination. Figure 1 shows the ROC curve for this model, illustrating the trade-off between sensitivity and specificity across classification thresholds.

**Fig. 2:**
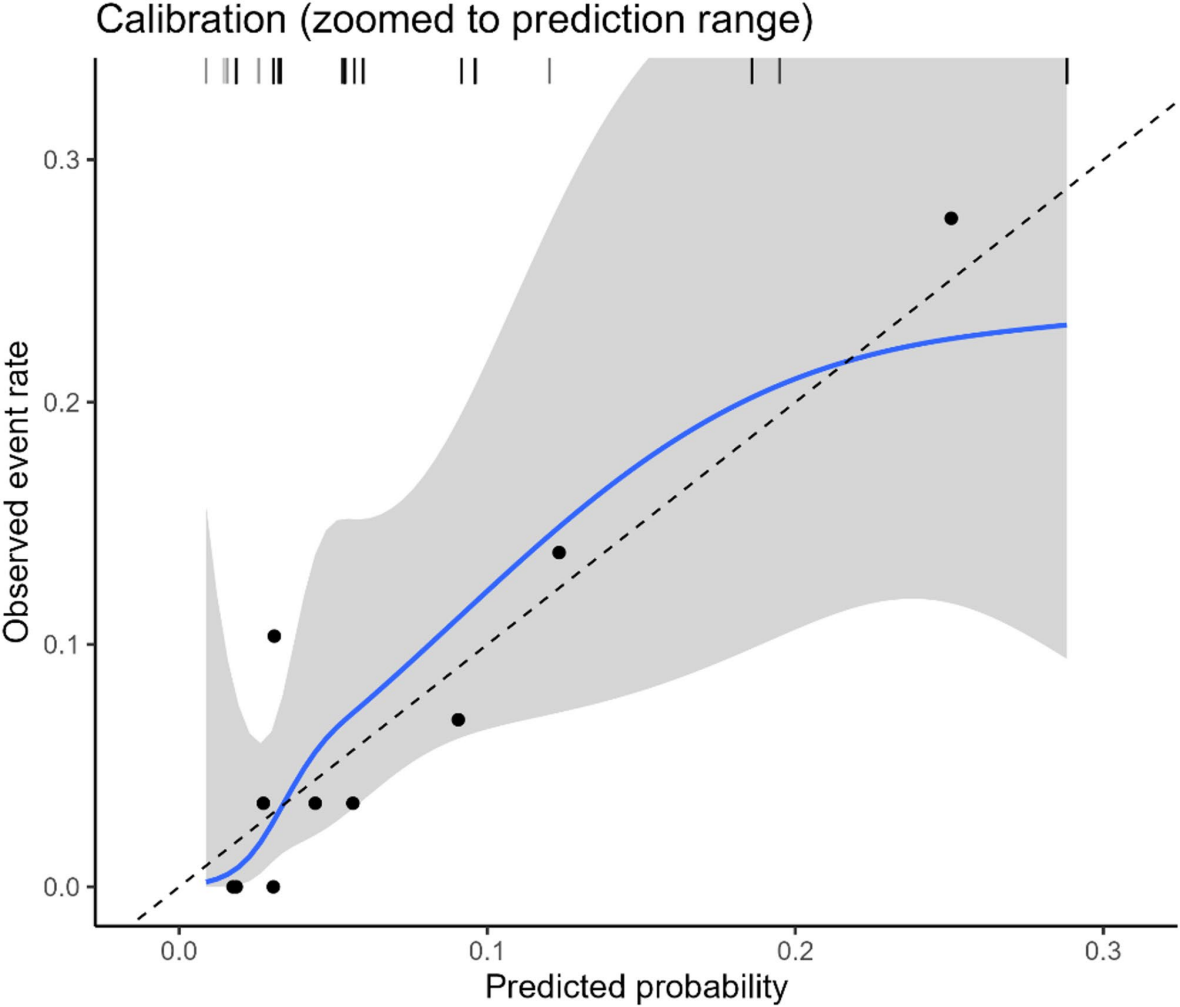
Calibration of the mixed-effects model using marginal predictions. The solid curve is a binomial smoother with 95% band; dots show decile-wise observed event rates; the dashed line indicates perfect calibration. Estimated intercept and slope are annotated (ideal: 0 and 1)

## Discussion

This study assessed the prevalence and associated predictors of irritable bowel syndrome (IBS) among medical students, a group known to be at elevated risk for stress-related disorders, in Ethiopia. The impetus for this research was the limited evidence on IBS in sub-Saharan African medical student populations, despite the unique academic and psychosocial stressors medical students face that may contribute to IBS.

Our findings indicate a prevalence of IBS of 6.89% among Ethiopian medical students, which is considerably lower than the prevalence reported in a previous Rome IV study among Gondar University medical students (23%) [17]. While the study from Gondar University was a preprint and has not yet been peer-reviewed thus far, it has been discussed here due to the lack of other studies on medical students in Ethiopia. The lower prevalence observed in our cohort, drawn from multiple universities across the country, may be explained by differences in participant demographics, environmental stressors, or academic workload.

While there is a paucity of studies reporting prevalence of IBS in African medical students using Rome IV criteria, an Egyptian study utilizing the Rome III criteria found a prevalence of 27.5% among 182 medical students [13]. The wide difference between our finding and that of this study is likely due to the Rome IV diagnostic criteria being more stringent than the Rome III [24], although the different demographic in the North African country may have played a role. A study conducted on a large sample of 424 medical students in the Sub-Saharan country of Benin (using the Rome IV), on the other hand, found a prevalence of 7.55%, which closely resembles our finding [25].

Our finding of a 6.89% prevalence is also lower than reported in many studies outside of African medical students using the Rome IV criteria, such as those from Peru (16.9%) and Pakistan (60%) [22, 26], but aligns with the pooled prevalence of 3.8% (95% CI 3·1–4·5; I2 = 96·6%) reported in a global meta-analysis [24]. The variation in prevalence in the different parts of the world could be due to socioeconomic and demographic factors that influence the occurrence of IBS.

In addition, our results show a significant association between IBS and anxiety, underscoring the role of psychosocial factors in the pathophysiology of IBS among medical students. This aligns with prior research, which identified emotional stress and anxiety as important risk factors for IBS [11, 12, 17]. This finding therefore highlights the need for ongoing mental health support and targeted interventions within medical training environments which will then reduce IBS symptoms and improve students’ overall well-being and academic performance. The observed odds ratio of 7.25 indicates that students with clinically relevant anxiety were substantially more likely to report IBS symptoms compared with their peers without anxiety. This finding stayed consistent across sensitivity analyses as anxiety showed a stable and reliable link with IBS even after accounting for differences between universities. The model’s AUC of 0.776 shows that psychological predictors clearly separate students with and without IBS. Given the intense stress of medical training, this association is plausible as anxiety could disrupt gut function and trigger IBS symptoms.

In both our study and the one conducted at Gondar University in Ethiopia [17], depression was not significantly associated with IBS after adjusting for anxiety and sex, although a significant association in the unadjusted model of our study likely reflected the overlap between anxiety and depression. The lack of significant association after adjustment is in contrast with findings in other countries, such as Egypt, Benin, Peru, and Pakistan [13, 22, 25, 26]. It is possible that limited statistical power in both Ethiopian studies may have played a role given the relatively small number of IBS cases. Low mental health literacy and stigmatization of mental health disorders, such as depression, in Ethiopia may have also resulted in lower reporting of depression [27].

Unlike findings from Western populations where IBS is more prevalent among females, our study did not demonstrate a significant difference between the sexes. This pattern is consistent with studies from Africa and parts of Asia, suggesting that the sex distribution of IBS may be influenced by genetic, environmental, or sociocultural factors specific to different regions [28]. Additionally, cultural perceptions and gender roles may influence how males and females recognize and report gastrointestinal symptoms [29, 30]. Stigma associated with discussing digestive or emotional problems may lead female students to underreport symptoms, and differences in healthcare-seeking behavior could contribute to the lack of apparent sex variation in our findings. The associations between IBS and other known risk factors, such as dietary habits, socioeconomic status, and family history, were not statistically significant in this study, possibly reflecting the relatively homogenous lifestyle and dietary patterns among Ethiopian medical students or the insufficient sample size to detect these associations [28]. These findings highlight important contextual factors in IBS expression among Ethiopian medical students.

This study has several strengths. It is the first multicenter study on the prevalence and predictors of irritable bowel syndrome among Ethiopian medical students, to the best of our knowledge. This therefore fills a significant data gap in Ethiopia and Sub-Saharan Africa. Another notable strength of this study is the use of internationally accredited diagnostic criteria and psychological assessment tools, namely Rome IV and HADS, which further strengthen the reliability of the findings. Additionally, the diversity of participants in the year of medical education and university location makes the sample more representative.

Despite these strengths, some limitations should be acknowledged. The number of IBS cases was relatively modest; however, it was sufficient to detect meaningful relationships (particularly with anxiety). Voluntary participation, promoted mainly through social media and university channels, may have introduced self-selection and healthy volunteer bias by attracting students who are more active online or health-conscious. Investigators at each university promoted the survey across different faculties and disciplines to encourage diverse participation, but such efforts cannot fully remove the risk of selection bias. Moreover, our data indicates that participant recruitment was not evenly distributed across universities, with a substantial proportion coming from Addis Ababa University. Although our analytical approach accounted for clustering by institution, this uneven representation may limit the generalizability of the results to medical students from other universities. The requirement for participants to complete every question ensured a fully populated dataset; however, it may have excluded individuals who did not feel comfortable or were unable to answer all items. Consequently, the study sample may not fully reflect the broader population. In addition, the exclusion of participants based on self-reported symptoms or family history relied solely on participants’ own accounts, which may not always be accurate and could have resulted in misclassification. Other alarm features were not assessed, which may have limited our ability to fully exclude organic causes. Between-university variance was estimated at the boundary, likely reflecting sparse events per university, which limits our ability to quantify clustering effects. Furthermore, some lifestyle factors, such as physical activity, were difficult to quantify precisely, and IBS subtypes were not specified. Collectively, these factors indicate that these findings should be interpreted with caution and future studies using more structured samples could strengthen the representatives and external validity of the results.

In conclusion, IBS is present among Ethiopian medical students, with anxiety emerging as a significant associated factor. The lower prevalence observed compared with that reported in previous local and international studies may reflect methodological and contextual differences. These findings underscore the importance of integrating psychosocial support into medical education to address both mental health and gastrointestinal symptoms among medical students. We believe that there is a strong need for multicenter and longitudinal research to clarify risk factors and develop effective preventive strategies in this population.

## Conclusion

Given its prevalence and impact on quality of life, relatively few data exist on epidemiology and risk factors for IBS at both the global and regional levels. Our findings highlight the potential influence of psychological factors on IBS among medical students in Ethiopia. However, selection bias may limit the generalizability of these findings to all medical students in Ethiopia. Further studies with larger and more diverse cohorts are recommended to better understand the psychosocial correlates of IBS in the medical student population.

## Supplementary Information

Supplementary Material 1

## Data Availability

The data collected and analyzed in this study are available from the corresponding author on reasonable request.

## Abbreviations

AUC: Area under the curve
CI: Confidence interval
HADS: Hospital anxiety and depression scale
HRQoL: health-related quality of life
IBS: Irritable bowel syndrome
OR: Odds ratio
