# Supplementary material for: Prevalence and associated factors of irritable bowel syndrome among medical students in Ethiopia: a cross-sectional study
Source: BMC Gastroenterol. 2025 Nov 26;25:886. doi: 10.1186/s12876-025-04415-8 (PMC12750862; doi:10.1186/s12876-025-04415-8)
Supplement: Supplementary file 1 — Supplementary Material 1 [file 12876_2025_4415_MOESM1_ESM.docx]

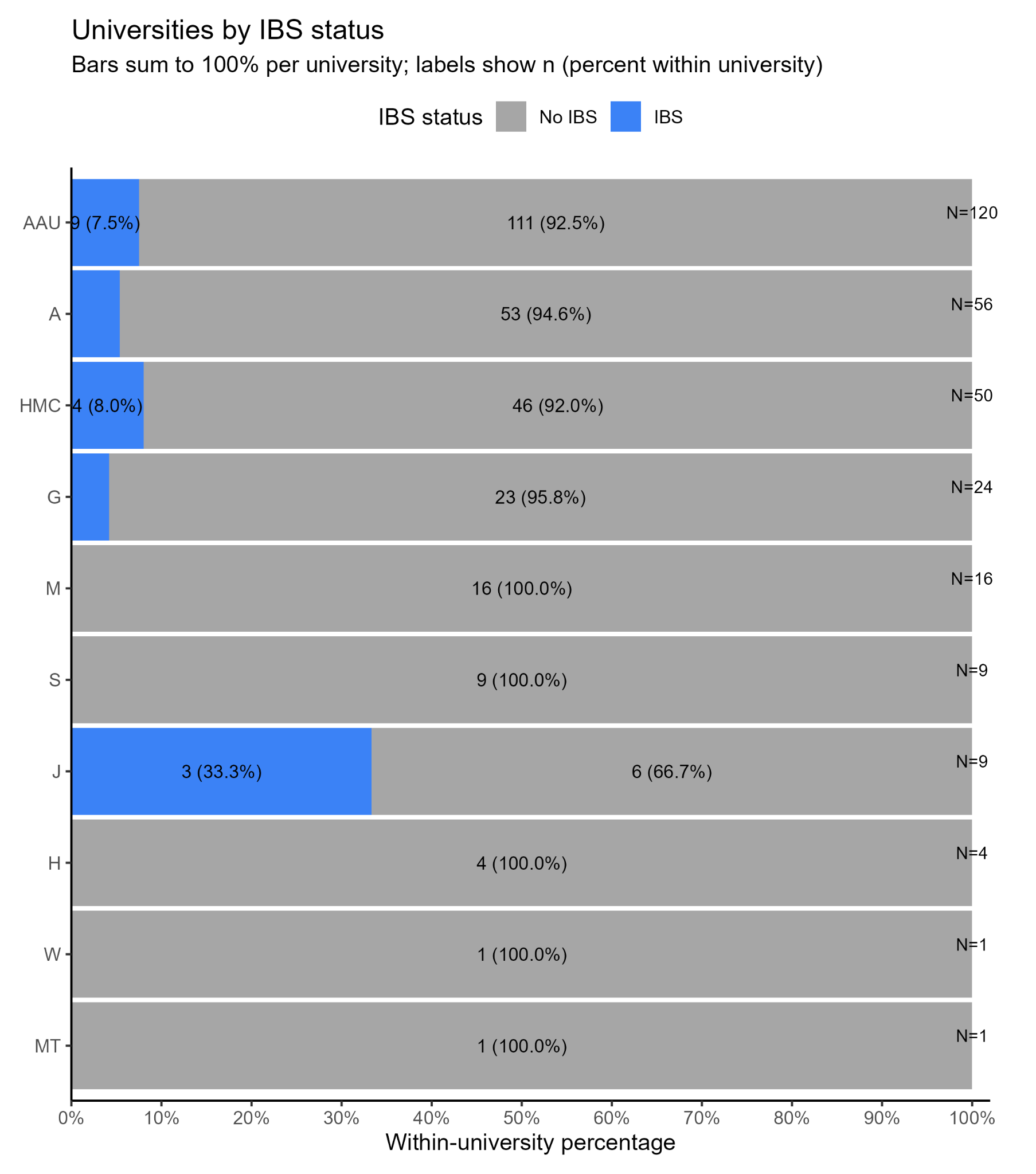


Figure 1 Distribution of IBS status by university (stacked 100% bars). Grey = No IBS, blue = IBS. Labels inside segments show n (percent within university); the total N for each university is shown at the right edge of the bar. Acronyms: AAU = Addis Ababa University; A = Ambo University; HMC = Hayat Medical College; G = University of Gondar; M = Mekelle University; S = St. Paul’s Hospital Millennium Medical College (SPHMMC); J = Jimma University; H = Hawassa University; W = Wollega University; MT = Mizan-Tepi University. Percentages for universities with very small N should be interpreted cautiously.
